# Etymologia: *Trypanosoma*

**DOI:** 10.3201/eid1209.ET1209

**Published:** 2006-09

**Keywords:** Etymologia,Trypanosoma, etymology

## [tri-pan′′o-so′mə]

From the Greek *trypanon*, “borer,” plus *sōma*, “body,” *Trypanosoma* is a genus of hemoflagellate protozoa, several species of which are pathogenic in humans. *Trypanosoma cruzi*, the etiologic agent of Chagas disease, is transmitted from its vector to humans in the insect’s feces, not its saliva, as is the case with most other arthropodborne organisms, including *Trypanosoma brucei*, the etiologic agent of sleeping sickness.

Sources: Dorland’s illustrated medical dictionary. 30th ed. Philadelphia: Saunders; 2003 and wikipedia.org

